# A taxonomic revision of the genus *Conidiobolus* (Ancylistaceae, Entomophthorales): four clades including three new genera

**DOI:** 10.3897/mycokeys.66.46575

**Published:** 2020-03-30

**Authors:** Yong Nie, De-Shui Yu, Cheng-Fang Wang, Xiao-Yong Liu, Bo Huang

**Affiliations:** 1 Anhui Provincial Key Laboratory for Microbial Pest Control, Anhui Agricultural University, Hefei 230036, China Anhui Agricultural University Hefei China; 2 School of Civil Engineering and Architecture, Anhui University of Technology, Ma’anshan 243002, China Anhui University of Technology Ma’anshan China; 3 State Key Laboratory of Mycology, Institute of Microbiology, Chinese Academy of Sciences, Beijing 100101, China Institute of Microbiology, Chinese Academy of Sciences Beijing China

**Keywords:** Zygomycetes, *
Entomophthorales
*, Morphology, Phylogeny, New taxa

## Abstract

The genus *Conidiobolus* is an important group in entomophthoroid fungi and is considered to be polyphyletic in recent molecular phylogenies. To re-evaluate and delimit this genus, multi-locus phylogenetic analyses were performed using the large and small subunits of nuclear ribosomal DNA (nucLSU and nucSSU), the small subunit of the mitochondrial ribosomal DNA (mtSSU) and the translation elongation factor 1-alpha (EF-1α). The results indicated that the *Conidiobolus* is not monophyletic, being grouped into a paraphyletic grade with four clades. Consequently, the well-known *Conidiobolus* is revised and three new genera *Capillidium*, *Microconidiobolus* and *Neoconidiobolus* are proposed along with one new record and 22 new combinations. In addition, the genus *Basidiobolus* is found to be basal to the other entomophthoroid taxa and the genus *Batkoa* locates in the *Entomophthoraceae* clade.

## Introduction

More than 250 species of entomophthoroid fungi were isolated from insects, soil and litter throughout the world (Gryganskyi et al. 2013). For a long time, this group has been considered to be polyphyletic (Nagahama et al. 1995; Jensen et al. 1998; James et al. 2006; Liu and Voigt 2010) and was classified into a subphylum *Entomophthoromycotina* and a pending taxon *Basidiobolus* (Hibbett et al. 2007). However, a recent phylogeny using the multi-gene dataset, 18S rDNA, 28S rDNA, mtSSU and RPB2, indicated that this group formed a monophyletic lineage including *Basidiobolus* and it was consequently reclassified as a new fungal phylum *Entomophthoromycota*. More recently, a phylogenomic analysis (192 clusters of orthologous proteins) has divided traditional zygomycotan into two phyla *Mucoromycota* and *Zoopagomycota* and the entomophthoroid fungi have been re-assigned into the subphylum *Entomophthoromycotina* under the latter phylum (Spatafora et al. 2016). This taxonomic scheme was supported by the phylogeny of mitochondrial genomes (Nie et al. 2019).

Together with other two genera *Ancylistes* and *Macrobiotophthora*, the genus *Conidiobolus* belongs to *Ancylistaceae*, *Entomophthorales*, *Entomophthoromycetes*, *Entomophthoromycotina* (Humber 2012). There are six and two accepted species within the *Ancylistes* and *Macrobiotophthora*, respectively, while *Conidiobolus*, one of the largest groups in the entomophthoroid fungi, contains 76 names (http://www.indexfungorum.org/). The genus *Conidiobolus* is typified by *C. utriculosus* Bref. 1884 and characterised morphologically by simple sporophores, globose to pyriform multinucleate primary conidia, various types of secondary conidia and resting spores (Brefeld 1884; Humber 1997). Up to the 1940s, for half a century, only three more species were reported, *C. minor* Bref., *C. villosus* Martin and *C. brefeldianus* Couch (Brefeld 1884; Martin 1925; Couch 1939). In the 1950s–1960s, 38 *Conidiobolus* species and a variety were isolated from the United States and India (Drechsler 1952, 1953a, b, 1954, 1955a, b, c, 1956, 1957a, b, c, 1960, 1961, 1962, 1965; Srinivasan and Thirumalachar 1961, 1962a, b, 1965, 1967, 1968a, b). Based on a numerical taxonomy, King (1976a, b, 1977) recognised 27 definitive species. Since then, along with some new combinations, 10 more species have been added to *Conidiobolus* (Bałazy et al. 1987; Waters and Callaghan 1989; Bałazy 1993; Huang et al. 2007; Waingankar et al. 2008; Nie et al. 2012, 2016, 2017, 2018). A total of 37 species are currently accepted in this genus (Nie et al. 2018).

Three subgenera – *Capillidium*, *Conidiobolus* and *Delacroixia* – were proposed within the *Conidiobolus*, based on shape of the secondary conidia and, amongst them, the subgenus Delacroixia was reduced from generic rank (Ben-Ze’ev and Kenneth 1982). This subgeneric criterion provided a valuable contribution for the taxonomy of the genus *Conidiobolus* (Humber 1989). Since the 1990s, molecular analysis has become an increasingly important tool for fungal taxonomy (Bruns et al. 1991; Taylor et al. 2000). The nucLSU rDNA and EF-1α regions proved to be distinguishable amongst *Conidiobolus* species (Nie et al. 2012), while nucSSU rDNA indicated the genus *Conidiobolus* might be a polyphyletic group (Jensen et al. 1998). The subgeneric circumscription was not defined because of instability to form a certain type of secondary conidia for each phylogenetic clade (Callaghan et al. 2000; Gryganskyi et al. 2013; Nie et al. 2018). Besides, the phylogenetic relationships amongst species of *Conidiobolus* have not been fully resolved due to the absence of types. The genus *Batkoa*, morphologically similar to *Conidiobolus*, was phylogenetically closely related to *Entomophthoraceae* rather than *Ancylistaceae* (Gryganskyi et al. 2012, 2013).

In the present study, a reclassification of the entomophthoroid fungi, including as many as available *Conidiobolus* types, was constructed based on four loci (nucSSU, nucLSU, EF-1α and mtSSU) to present the taxonomic delimitation of the genus *Conidiobolus* and to re-evaluate the phylogenetic relationship between *Basidiobolus* and *Batkoa*.

## Materials and methods

### Isolates and morphology

A total of 26 ex-types of *Conidiobolus* were purchased from the American Type Culture Collection, Manassas, USA (ATCC) and collected from the China General Microbiological Culture Collection Center, Beijing, China (CGMCC) and the Research Center for Entomogenous Fungi of Anhui Agricultural University, Anhui Province, China (RCEF). Dried cultures were deposited in the Herbarium Mycologicum Academiae Sinicae, Beijing, China (HMAS). Morphology was observed with an Olympus BX51 research microscope and photographed by an Olympus DP25 microscope-camera system. Growth diameter on PDA (potato 200 g, dextrose 20 g, agar 20 g, H_2_O 1 l), Mycelia, primary conidiophores, primary conidia, microconidia, capilliconidia and resting spores were measured and described with the method of King (1976a).

### DNA extraction, PCR amplification and sequencing

Fungal strains were incubated on PDA for 7 d at 21 °C. Total genomic DNA was extracted from the fresh fungal mycelia by using modified CTAB method (Watanabe et al. 2010). Four gene portions from cell nuclei and mitochondria and one protein coding gene were used in this study: the large subunit of nuclear ribosomal RNA genes (nucLSU), the small subunit of nuclear ribosomal RNA genes (nucSSU), the small subunit of mitochondrial ribosomal RNA genes (mtSSU) and the translation elongation factor 1-alpha gene (EF-1α). The nucLSU region was amplified with the primers LR0R and LR5 (Vilgalys and Hester 1990), the nucSSU region with nucSSU-0021-5’ (Gargas and DePriest 1996) and nucSSU-1780-3’ (DePriest 1993) and EF-1α region with the primers EF983 and EF1aZ-1R (http://www.aftol.org/primers.php). These PCR reactions have been described by Liu et al. (2005), Jensen et al. (1998) and Nie et al. (2012). The primers used for the mtSSU region were mtSSU1 and mtSSU2R and the PCR reaction was performed using the following cycling parameters: denaturation at 94 °C for 3 min, followed by 35 cycles of denaturation at 94 °C for 1 min, annealing at 52 °C for 1 min, extension at 72 °C for 1 min and finalised with an extra extension at 72 °C for 7 min (Zoller et al. 1999). PCR products were purified and sequenced by Shanghai Genecore Biotechnologies Company (Shanghai, China) with the same primers as relative PCR. The nucleotide sequence data have been deposited in the GenBank (Table 1).

**Table 1.** The species used in phylogenetic analyses.

Species	Strains*	GenBank accession numbers			
		nucSSU	nucLSU	EF-1α	mtSSU
*Allomyces arbusculus*	AFTOL 300	AY552524	DQ273806	DQ275334	–
*Basidiobolus haptosporus*	ARSEF 261	JX242606	JX242586	–	JX242626
*B. heterosporus*	CBS 311.66	JX242607	JX242587	–	JX242627
*B. magnus*	CBS 205.64	JX242608	JX242588	–	JX242628
*B. meristosporus*	CBS 931.73	JX242609	JX242589	–	JX242629
*B. microsporus*	CBS 130.62 (T)	JX242610	JX242590	–	JX242630
*B. ranarum*	NRRL 34594	AY635841	DQ273807	DQ275340	EF392490
*Batkoa apiculata*	ARSEF 3130	DQ177437	EF392404	–	EF392513
*B. gigantea*	ARSEF 214	JX242611	JX242591	–	JX242631
*B. major*	ARSEF 2936	EF392559	EF392401	–	EF392511
B. obscurus**	CBS 182.60	JX242614	JX242595	–	JX242635
B. pseudapiculata**	ARSEF 395	EF392557	EF392378	–	EF392508
*Coemansia reversa*	AFTOL 140	AY546685	AY546689	DQ282615	–
*Conidiobolus adiaeretus*	ARSEF 451 (T)	–	KC461182	–	–
*C. adiaeretus*	CGMCC 3.15888	–	MN061284	MN061481	MN061287
*C. antarcticus*	ARSEF 6913 (T)	–	DQ364207	–	DQ364227
*C. bangalorensis*	ARSEF 449 (T)	–	DQ364204	–	DQ364225
*C. brefeldianus*	ARSEF 452 (T)	AF368506	EF392382	–	EF392495
*C. chlamydosporus*	ATCC 12242 (T)	–	JF816212	JF816234	MK301178
*C. coronatus*	NRRL 28638	AF113418	AY546691	DQ275337	–
*C. coronatus*	RCEF 4518	–	JN131537	JN131543	–
*C. couchii*	ATCC 18152 (T)	–	JN131538	JN131544	MK301179
*C. dabieshanensis*	CGMCC 3.15763 (T)	–	KY398125	KY402206	MK301180
*C. denaeosporus*	ATCC 12940 (T)	–	JF816215	JF816228	MK301181
*C. firmipilleus*	ARSEF 6384	JX242612	JX242592	–	JX242632
*C. gonimodes*	ATCC 14445 (T)	–	JF816221	JF816226	MK301182
*C. heterosporus*	RCEF 4430	–	JF816225	JF816239	MK301183
*C. humicolus*	ATCC 28849 (T)	–	JF816220	JF816231	MK301184
*C. incongruus*	NRRL 28636	AF113419	AF113457	–	–
*C. iuxtagenitus*	ARSEF 6378 (T)	–	KC788410	–	–
*C. iuxtagenitus*	RCEF 4445	–	JX946695	JX946700	MK333391
*C. khandalensis*	ATCC 15162 (T)	–	KX686994	KY402204	MK301185
*C. lachnodes*	ARSEF 700	–	KC788408	–	–
*C. lamprauges*	ARSEF 2338	AF296754	DQ364206	–	DQ364226
*C. lichenicolus*	ATCC 16200 (T)	–	JF816216	JF816232	MK301186
*C. lobatus*	ATCC 18153 (T)	–	JF816218	JF816233	MK301187
*C. marcosporus*	ATCC 16578 (T)	–	KY398124	KY402209	MK301188
*C. megalotocus*	ATCC 28854 (T)	–	MF616383	MF616385	MK301189
*C. mirabilis*	CGMCC 3.17763 (T)	–	MH282852	MH282853	MK333389
*C. mycophagus*	ATCC 16201 (T)	–	JX946694	JX946698	MK301190
*C. mycophilus*	ATCC 16199 (T)	–	KX686995	KY402205	MK301191
*C. nodosus*	ATCC 16577 (T)	–	JF816217	JF816235	MK333388
*C. osmodes*	ARSEF 79	AF368510	EF392371	–	DQ364219
*C. osmodes*	RCEF4447	–	JN131539	JN131545	MK333392
*C. pachyzygosporus*	CGMCC 3.17764 (T)	–	KP218521	KP218524	MK333390
*C. paulus*	ARSEF 450 (T)	–	vv	–	–
*C. polyspermus*	ATCC 14444 (T)	–	MF616382	MF616384	MK301193
*C. polytocus*	ATCC 12244 (T)	–	JF816213	JF816227	MK301194
*C. pumilus*	ARSEF 453 (T)	JX242615	EF392383	–	EF392496
*C. rhysosporus*	ATCC 12588 (T)	–	JN131540	JN131546	MK301195
*C. sinensis*	RCEF 4952 (T)	–	JF816224	JF816238	MK301196
*C. stilbeus*	RCEF 5584 (T)	–	KP218522	KP218525	MK301197
*C. stromoideus*	ATCC 15430 (T)	–	JF816219	JF816229	MK301198
*C. terrestris*	ATCC 16198 (T)	–	KX752050	KY402208	MK301199
*C. thromboides*	ATCC 12587 (T)	–	JF816214	JF816230	MK301200
*C. thromboides*	FSU 785	JX242616	JX242597	–	JX242637
*C. thromboides*	RCEF 4492	–	JF816223	JF816236	MK333393
*C. undulatus*	ATCC 12943 (T)	–	JX946693	JX946699	MK301201
*Dimargaris bacillispora*	AFTOL 136	AB016020	DQ273791	DQ282609	–
*Endogone pisiformis*	AFTOL 539	DQ322628	DQ273811	DQ282618	–
*Entomophaga aulicae*	ARSEF 172	EF392542	EF392372	–	EF392487
*E. conglomerata*	ARSEF 2273	AF368509	–	–	–
*E. maimaga*	ARSEF 1400	EF392556	EF392395	–	EF392505
*Eryniopsis caroloniana*	ARSEF 640	EF392552	EF392387	–	EF392500
*Entomophthora chromaphidis*	ARSEF 1860	AF353725	–	–	–
*E. culicis*	ARSEF 387	AF368516	–	–	–
*E. grandis*	ARSEF 6701	–	DQ481229	–	–
*E. scatophaga*	ARSEF 6704	–	DQ481226	–	–
*E. muscae*	ARSEF 3074	AY635820	DQ273772	DQ275343	–
*E. planconiana*	ARSEF 6252	AF353723	GQ285878	–	–
*E. schizophorae*	ARSEF 5348	AF052402	GQ285883	–	–
*E. syrphi*	ARSEF 5595	–	DQ481230	–	–
*E. tripidium*	ARSEF 6518	AF296755	–	–	–
*Erynia conica*	ARSEF 1439	AF368513	EF392396	–	EF392506
*E. ovispora*	ARSEF 400	JX242620	JX242601	–	JX242641
*E. rhizospora*	ARSEF 1441	AF368514	EF392397	–	EF392507
*E. sciarae*	ARSEF 1870	AF368515	EF392399	–	EF392509
*Furia americana*	ARSEF 742	EF392554	EF392389	–	–
*F. gastropachae*	ARSEF 5541	EF392562	EF392407	–	EF392516
*F. ithacensis*	ARSEF 663	EF392553	EF392388	–	EF392501
*F. neopyralidarum*	ARSEF 1145	AF368518	EF392394	–	EF392504
*F. pieris*	ARSEF 781	AF368519	EF392390	–	EF392502
*F. virescens*	ARSEF 1129	EF392555	EF392393	–	EF392503
*Gaertneriomyces semiglobiferus*	AFTOL 34	AF164247	DQ273778	DQ275338	–
*Macrobiotophthora vermicola*	ARSEF 650	AF052400	–	–	–
*Massospora cicadina*	ARSEF 374	EF392548	EF392377	–	EF392492
*Mortierella verticillata*	AFTOL 141	AF157145	DQ273794	–	–
*Pandora blunckii*	ARSEF 217 (T)	JX242621	JX242602	–	–
*P. delphacis*	ARSEF 459	AF368521	EF392384	–	EF392497
*P. dipterigena*	ARSEF 397	AF368522	EF392380	–	EF392565
*P. kondoiensis*	CBS 642.92	JX242622	JX242603	–	JX242642
*P. neoaphidis*	ARSEF 3240	EF392560	EF392405	–	EF392514
*Piptocephalis corymbifera*	AFTOL 145	AB016023	AY546690	DQ282619	–
*Rhizophagus intraradices*	AFTOL 845	DQ322630	FJ461839	DQ282611	–
*Rozella allomycis*	AFTOL 297	AY635838	DQ273803	DQ275342	–
*Schizangiella serpentis*	ARSEF 2237	AF368523	EF392428	–	EF392488
*Strongwellsea castrans*	–	AF052406	–	–	–
*Zancudomyces culisetae*	AFTOL 29	AF277007	DQ273773	–	–
*Zoophthora anglica*	ARSEF 396	–	EF392379	–	EF392493
*Z. lanceolata*	ARSEF 469	EF392550	EF392385	–	EF392498
*Z. phalloides*	ARSEF 2281	EF392558	EF392400	–	EF392510
*Z. radicans*	ARSEF 388	JX242624	JX242605	–	JX242644

* AFTOL, Assembling the Fungal Tree of Life; ARSEF, ARS Entomopathogenic Fungus Collection (Ithaca, U.S.A.); ATCC, American Type Culture Collection (Manassas, U.S.A); CGMCC, China General Microbiological Culture Collection Center (Beijing, China); FSU, Jena Microbial Resource Collection (Friedrich-Schiller-University of Jena, Germany); NRRL, ARS Culture Collection (Peoria, U.S.A); RCEF, Research Center for Entomogenous Fungi (Hefei, China). T = ex-type. **Batkoa sp. CBS 182.60 was received as *Conidiobolus obscurus*, while *B. pseudapiculata*ARSEF 395 was received as *C. pseudapiculatus*.

### Phylogenetic analyses

More available nucLSU, nucSSU, mtSSU and EF-1α sequences of 14 *Conidiobolus* species and 47 other entomophthoroid fungi were obtained from GenBank. Ten species of *Glomeromycotina*, *Mortierellomycotina*, *Mucoromycotina*, *Kickxellomycotina*, *Zoopagomycotina*, *Blastocladiomycota*, *Chytridiomycota* and *Cryptomycota*, were chosen as outgroups. Alignments were constructed separately for each locus with MUSCLE 3.8.31 (Edgar 2004) and the concatenated matrices were assembled by SequenceMatrix 1.7.8 (Vaidya et al. 2011). The best model for the phylogenetic analysis was selected with Akaike Information Criterion (AIC) by using Modeltest 3.7 (Posada and Crandall 1998). Phylogenetic relationships were inferred using Maximum Likelihood (ML) and Bayesian Inference (BI). The best-scoring ML tree analysis was performed using raxmlGUI 1.5b1 with GTRGAMMA model and 1000 replicates (Silvestro and Michalak 2012). The BI analysis was performed using MrBayes 3.2.2 (Ronquist and Huelsenbeck 2003). Markov Chain Monte Carlo (MCMC) chains ran until the convergences met and the standard deviation fell below 0.01. The first 25% of trees were discarded as burn-in. The combined dataset was deposited at TreeBase (No. S25064). Phylogenetic trees were checked and modified in FigTree 1.4 (Rambaut 2012).

## Results

### Phylogenetic analyses

The combined dataset contained 4521 characters of nucLSU (1–1326), nucSSU (1327–3424), EF-1α (3425–4062) and mtSSU (4063–4521) after alignment. With the optimal model GTR+I+G and random starting trees, four Markov chains were run for 7 million generations and every 100^th^ generation was sampled once. ML and BI analyses of the combined dataset resulted in phylogenetic reconstructions with almost similar topologies and the average standard deviation of split frequencies was 0.006721 (BI).

**Figure 1.**
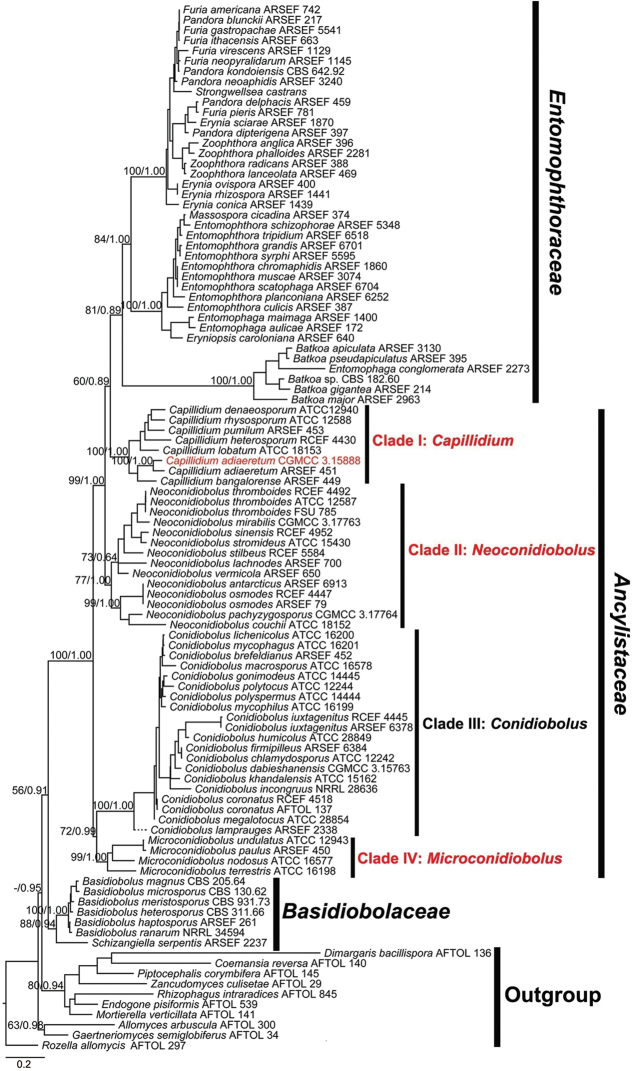
Phylogenetic tree constructed by maximum likelihood analyses of nucLSU, nucSSU, EF-1α and mtSSU sequences for *Entomophthoromycotina*, with some chytrid and mucoralean fungi as outgroups. Three new genera and one Chinese new record are shown in red. Maximum likelihood bootstrap values (≥ 50%) / Bayesian posterior probabilities (≥ 0.50) of main clades are indicated along branches. Scale bar indicates substitutions per site.

In the ML phylogenetic tree (Figure 1), the *Basidiobolaceae* lineage (88/0.94) is located at the base of the entomophthoroid fungi and is closely related to the *Ancylistaceae* group (56/0.91). The *Batkoa* lineage is grouped within the *Entomophthoraceae* Clade (60/0.89). All *Conidiobolus* lineages are clustered into a paraphyletic grade and therefore cannot be considered congeneric. Moreover, the *Conidiobolus* grade consists of four well supported clades. In detail, there are 7, 10, 16 and 3 species in Clade I (100/1.00), II (77/1.00), III (100/1.00) and IV (99/1.00), respectively.

### Taxonomy

In order to provide a more natural taxonomic classification, four genera (*Capillidium*, *Conidiobolus*, *Microconidiobolus* and *Neoconidiobolus*) and their type species (*Ca. heterosporum*, *C. utriculosus*, *M. paulus* and *N. thromboides*) are described here in this paper. Additionally, a new record *Ca. adiaeretum*, *C. coronatus* and *C. iuxtagenitus* with new isolates from China and *C. khandalensis* being first reported to produce microconidia are illustrated herein.

#### Capillidium

Taxon classificationFungiEntomophthoralesAncylistaceae

B. Huang & Y. Nie gen. nov.

D46F46A3-E0E1-5C9B-AD08-2A5C10757790

831596

##### Etymology.

Referring to unique ellipsoidal secondary conidia (capilliconidia).

##### Type species.

*Capillidium heterosporum* (Drechsler) B. Huang & Y. Nie.

##### Description.

Mycelia colourless. Primary conidiophores simple, bearing a single primary conidia. Primary conidia forcibly discharged multinucleate, colourless, globose, pyriform to obovoid. Two kinds of replicative conidia, the first one is similar and smaller than primary conidia, the second one (capilliconidia) arises from elongate and slender conidiophores. Zygospores present or absent, formed in axial alignment with conjugating segments, globose to subglobose, often smooth, sometimes rough, colourless or yellowish.

##### Notes.

Conidiobolus subgen. Capillidium Ben-Ze’ev & Kenneth was firstly established to include species with capilliconidia (Ben-Ze’ev and Kenneth 1982). In this phylogenetic analysis, all members of the subgenus Capillidium grouped with good support (100/1.00) and, therefore, it was raised from subgenus to genus status based on the monophyly, as well as the stability to form ellipsoidal secondary conidia (capilliconidia). In addition to capilliconidia, *C. adiaeretus* also produces microconidia.

#### Capillidium heterosporum

Taxon classificationFungiEntomophthoralesAncylistaceae

(Drechsler) B. Huang & Y. Nie comb. nov.

033D89FA-FE07-5B76-ABF5-D805C655AA30

831601

Figure 2

Conidiobolus heterosporus Drechsler, Am. J. Bot. 40: 107 (1953). Basionym.
=Conidiobolus rugosus Drechsler, Am. J. Bot. 42: 437 (1955).

##### Specimens examined.

China, Anhui Province, Plant detritus, 8 Nov 2008, *C.F. Wang, RCEF 4430*.

##### Description.

Colonies on PDA at 25 °C after 3 d, white, reaching ca. 21 mm in diameter. Mycelia colourless, 5–9 μm wide. Primary conidiophores, colourless, unbranched and producing a single globose conidium with widening upwards, extending to a length of 30–245 μm into the air, 8–17 μm wide. Primary conidia forcibly discharged, colourless, globose to subglobose, measuring 12–37 μm in greatest length and 11–31 μm in total width, including a basal papilla 1.5–5 μm high and 5–12 μm wide. After discharging on to 2% water-agar, similar and smaller secondary conidia arise from primary conidia, 1–6 ellipsoidal secondary conidia (capilliconidia, 10–20 × 12–38 μm) arise from slender conidiophores (50–250 × 2.5–4 μm). Resting spores not observed.

##### Notes.

The ex-type living culture is ATCC 12941 (United States, Maryland, 18 Mar 1952, Drechsler).

**Figure 2.**
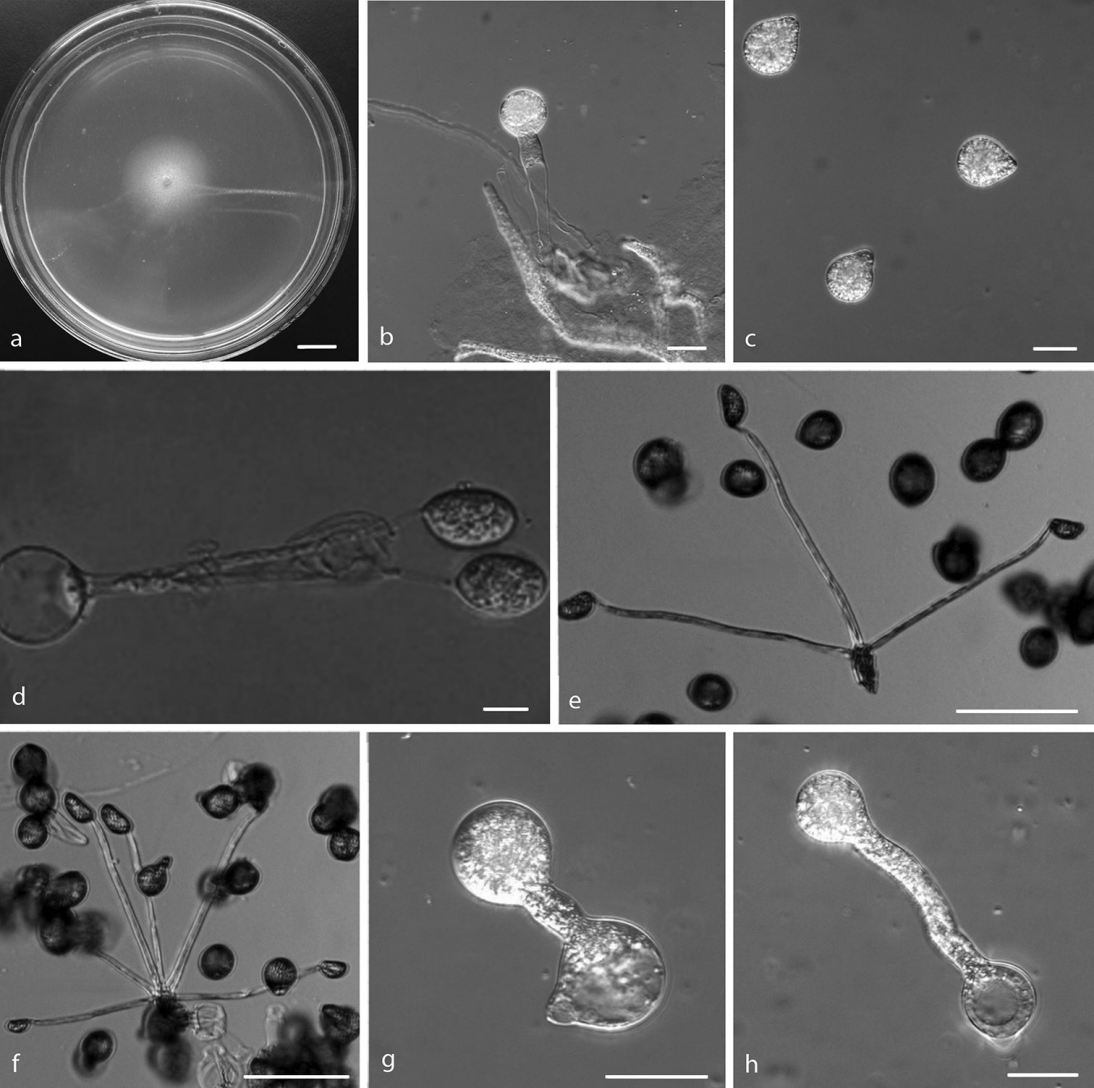
*Capillidium heterosporum***a** colony on PDA after 3 d at 25 °C **b** primary conidiophores bearing primary conidia **c** primary conidia **d, e, f** ellipsoidal secondary conidia arising from slender conidiophores **g, h** production of secondary conidia. Scale bars: 10 mm (**a**); 20 μm (**b, c, d, g, h**); 100 μm (**e, f**).

#### Capillidium adiaeretum

Taxon classificationFungiEntomophthoralesAncylistaceae

(Drechsler) B. Huang & Y. Nie comb. nov.

348681A3-0E38-5892-8481-DEEAF76FC24A

831602

Figure 3

Conidiobolus adiaeretus Drechsler, J. Wash. Acad. Sci. 43: 42 (1953). Basionym.

##### Specimens examined.

China, Jiangsu Province, Nanjing City, Laoshan Forest Park, 32°5'58"N, 118°35'53"E, Plant detritus, 1 Dec 2018, *Y. Nie and Y. Gao, HMAS 248358*, culture *CGMCC 3.15888 (=RCEF 6550)*.

##### Description.

Colonies on PDA at 25 °C after 3 d, white, reaching ca. 7–10 mm in diameter. Mycelia colourless, 3–4.5 μm wide. Primary conidiophores, colourless, unbranched and producing a single globose conidium with widening upwards; they offer a pronounced dimensional contrast with the mycelial filaments, extending to a length of 50–210 μm into the air, 3–25 μm wide. Primary conidia forcibly discharged, colourless, globose, measuring 15–45 μm in greatest length and 13–42 μm in total width, including a basal papilla 2–6 μm high and 5–17 μm wide. After discharging on to 2% water-agar, similar and smaller secondary conidia arise from primary conidia, two generations of multiple spherical units forming on the parent globose conidia Microconidia only formed from the second set, 5–12 × 9–10 μm. Capilliconidia formed readily from discharged microconidia, 16–24 × 5–6 μm. Chlamydospores formed within the substratum, colourless, globose to ellipsoidal, 13–40 × 15–45 μm.

##### Notes.

The species was firstly reported from America (Drechsler 1953a). The ex-type living culture is ATCC 12589 isolated by Drechsler (1953a). It is mainly characterised and differs from other *Capillidium* species by its ability to form both microconidia and capilliconidia (Callaghan et al. 2000). The Chinese specimen CGMCC 3.15888 clusters completely (100/1.00) with an isotype ARSEF 451 (98% sequence similarity in nucLSU) and fits well with its morphological descriptions. It is reported in China for the first time.

**Figure 3.**
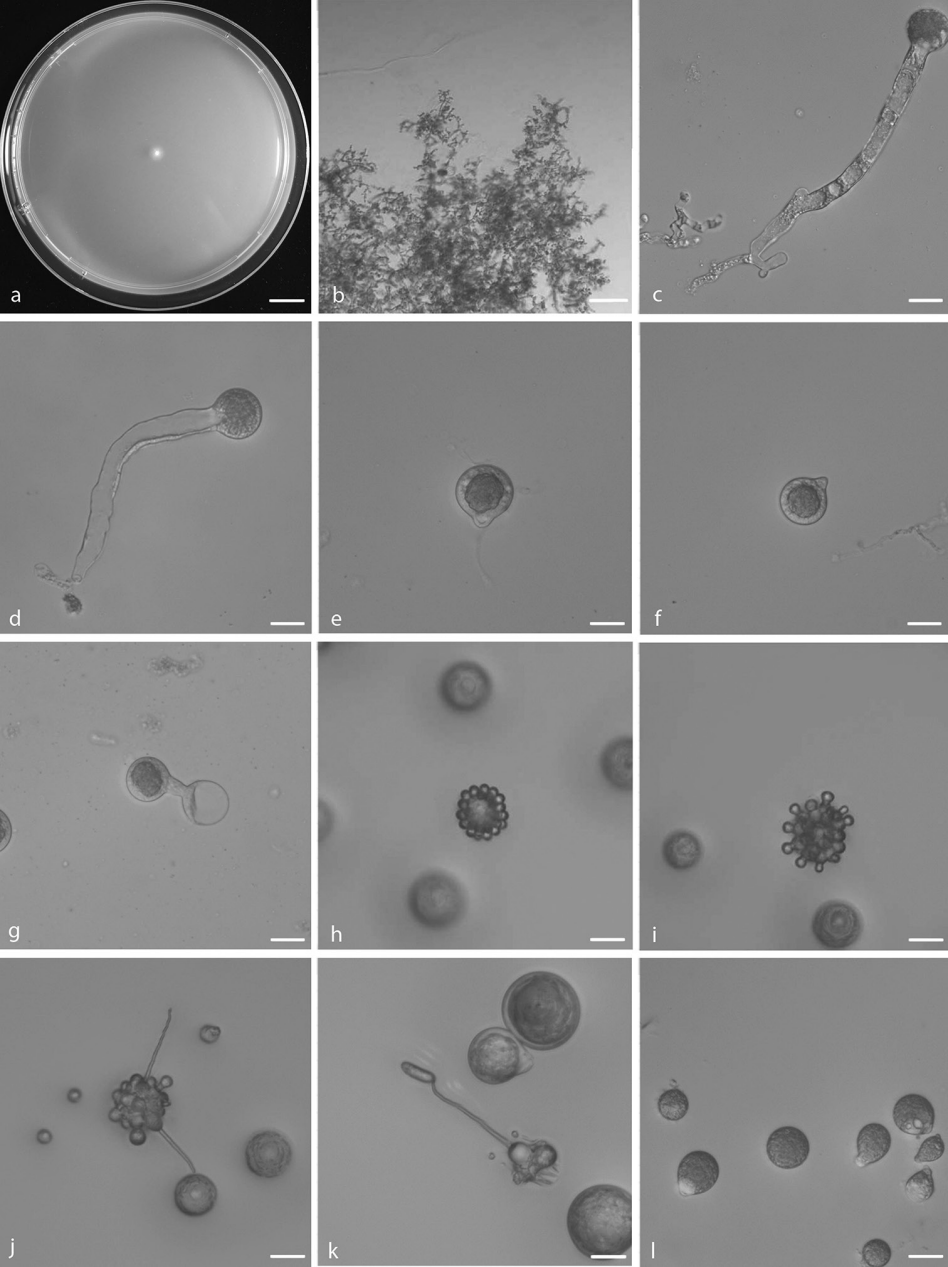
*Capillidium adiaeretum***a** colony on PDA after 3 d at 25 °C **b** mycelia **c, d** primary conidiophores bearing primary conidia **e, f** primary conidia **g** Production of secondary conidia **h** first stage of forming microconidia **i** second stage of forming microconidia **j, k** ellipsoidal secondary conidia arising from slender conidiophores **l** chlamydospores. Scale bars: 10 mm (**a**); 100 μm (**b**); 20 μm (**c–l**).

#### Conidiobolus

Taxon classificationFungiEntomophthoralesAncylistaceae

Bref., Mykol. Untersuch. 6(2): 35 (1884), emend.

A6638E2E-FF16-53E3-B738-84069BF5E7DF

20144

= Delacroixia Sacc. & P. Syd., Syll. fung. (Abellini) 14(1): 457 (1899).
Conidiobolus subgen. Delacroixia (Sacc. & P. Syd.) Tyrrell & Macleod, J. Invert. Pathol. 20: 12 (1972).

##### Type species.

*Conidiobolus utriculosus* Bref.

##### Description.

Mycelia colourless. Primary conidiophores simple or branched dichotomously, positively phototropic, bearing a single or 2–4 primary conidia. Primary conidia forcibly discharged, multinucleate, colourless, pyriform, obovoid, globose to subglobose. Secondary conidia usually with shape of primary conidia but smaller, formed singly on short secondary conidiophores. Microspores arising from primary or secondary conidia. Villose appendaged globose conidia and formed villose conidia. Chlamydospores formed intercalarily within assimilative hyphae. Zygospores formed in axial alignment with one or two (homothallic or heterothallic) conjugating segments.

##### Notes.

*C. utriculosus*, the type species of the genus *Conidiobolus*, has not been re-collected since Brefeld isolated it in 1884 and most taxonomists working on entomophthoroid fungi now universally recognised it as *C. coronatus* (Gryganskyi et al. 2013). However, the smaller pear-shaped conidia of *C. utriculosus* are different from the larger globose conidia of *C. coronatus* and villose spores in *C. coronatus* are not observed in *C. utriculosus* (Brefeld 1884; King 1977). Consequently, *C. coronatus* is not synonymised with *C. utriculosus* in this study. Instead, this study agrees with Srinivasan and Thirumalachar (1967) and King (1977) to place *C. minor* in synonymy with *C. utriculosus* because the small conidia of *C. minor* were probably replicative conidia of *C. utriculosus*. Nevertheless, neither *C. utriculosus* nor *C. minor* has available living cultures. Therefore, we have not yet designated an epitype and thus no DNA sequences for explaining this type. Fortunately, we are able to recognise clade III (Fig. 1) as *Conidiobolus* on the basis of its synapomorph, namely microspores.

#### Conidiobolus utriculosus

Taxon classificationFungiEntomophthoralesAncylistaceae

Bref., Mykol. Untersuch. 6(2): 35 (1884)

BAD66584-64D0-538B-90BE-223F06941DD7

144259 (MBT391377)

= Conidiobolus minor Bref., Mykol. Untersuch. 6(2): 35, 68 (1884).

##### Specimens examined.

No ex-type.

##### Description.

Refer to Brefeld (1884) and King (1977).

##### Notes.

Due to the lack of ex-type, plates 3, 4, and 5 in Brefeld, Mykol. Untersuch. 6(2): 35 (1884) are designated here as the lectotype for *Conidiobolus utriculosus*.

#### Conidiobolus coronatus

Taxon classificationFungiEntomophthoralesAncylistaceae

(Costantin) A. Batko, Entomophaga, Mémoires hors série 2: 129 (1964)

C501E77E-6761-514E-8E81-5DC66636ED0F

283037

Figure 4

Boudierella coronata Costantin, Bull. Soc. mycol. Fr. 13: 40 (1897). Basionym.
Delacroixia coronata (Costantin) Sacc. & P. Syd., Syll. fung. (Abellini) 14(1): 457 (1899).
Entomophthora coronata (Costantin) Kevorkian, J. Agric. Univ. Puerto Rico 21(2): 191 (1937).
= Conidiobolus villosus G.W. Martin, Bot. Gaz. 80(3): 317 (1925).

##### Specimens examined.

China, Shandong Province, Plant detritus, 20 Mar 2009, *C.F. Wang, RCEF 4518*.

##### Description.

Colonies grown on PDA for 3 d at 21 °C, reaching ca. 65 mm in diameter. Mycelia colourless, 8–20 μm wide. Primary conidiophores, positively phototropic, colourless, unbranched and producing a single globose conidium, extending to a length of 53–287 μm into the air, 7.5–20.5 μm wide. Primary conidia forcibly discharged, colourless, globose, measuring 36–52 μm in greatest width and 42–65 μm in total length, including a basal papilla 12–18 μm high and 6.5–14 μm wide. After discharging on to 2% water-agar, similar and smaller secondary conidia arise from primary conidia. Microconidia produced readily from primary conidia, globose or almond-shaped, 13–19 × 11–15 μm. Villose spores formed after 4–5 d, globose, 20–42 μm.

##### Notes.

The ex-type living culture is ATCC 28691 (United States, Louisiana, Plant detritus, 3 January 1972). Due to the absence of molecular data of ex-type strain ATCC 28691, the molecular data of the authentic strain NRRL 28638, which has been applied in many other phylogenetic analysis (James et al. 2006; Liu and Voigt 2011; Gryganskyi et al. 2012; Tretter et al. 2014; Spatafora et al. 2016), was used in this study instead. The monotypic genus *Delacroixia* was typified by *D. coronata* which was transferred from an ascomycete *Boudierella coronata* Costantin (Costantin 1897; Saccardo and Sydow 1899). After that, it was reclassified as a subgenus of *Conidiobolus*, namely *Conidiobolus* sub. *Delacroixia* (Sacc. & P. Syd.) Tyrrell & MacLeod to define all those *Conidiobolus* species capable of forming microspores and, consequently, *D. coronata* was recombined as *C. coronatus* (Tyrrell and MacLeod 1972; Ben-Ze’ev and Kenneth 1982).

**Figure 4.**
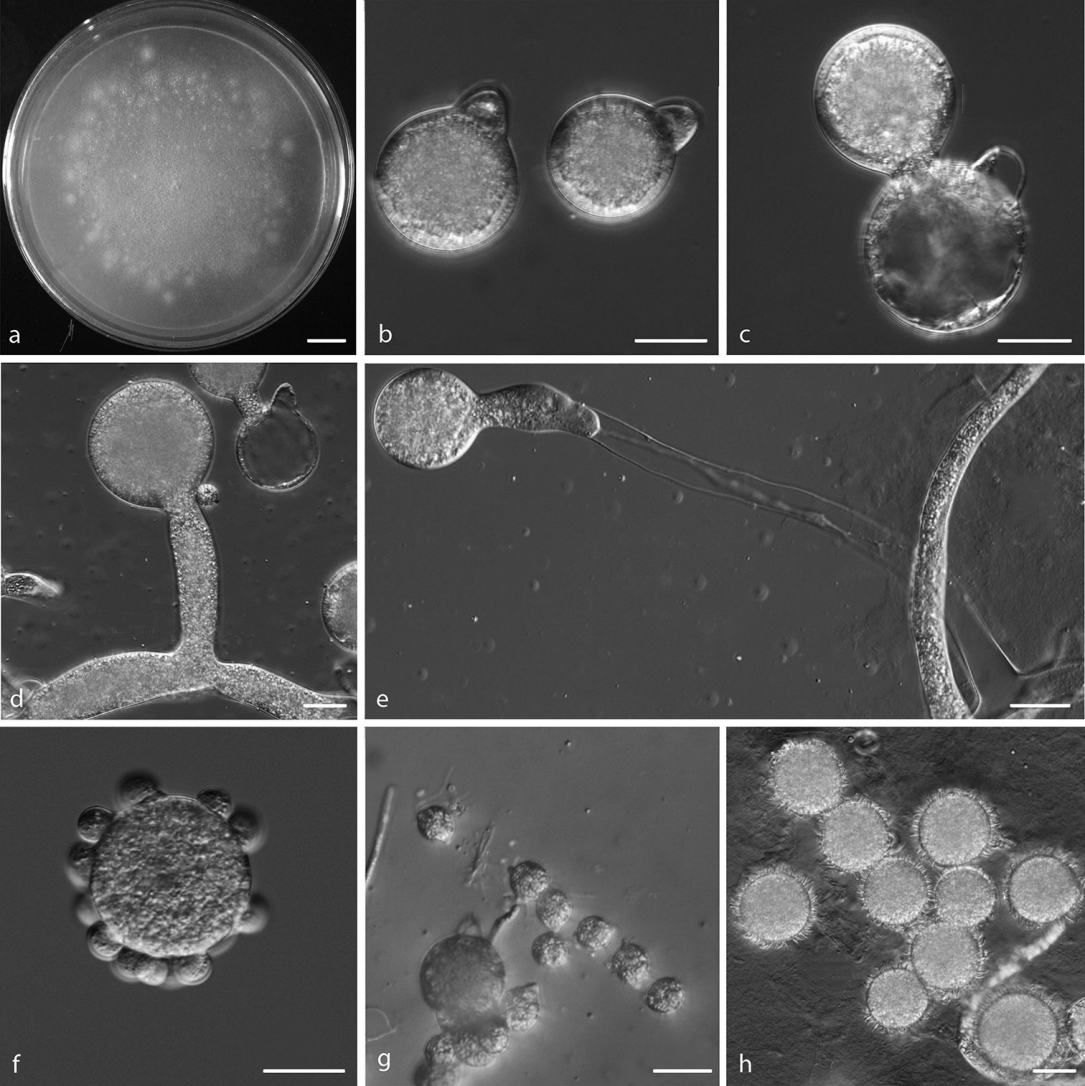
*Conidiobolus coronatus***a** colony on PDA after 3 d at 21 °C **b** primary conidia **c** production of secondary conidia **d, e** primary conidiophores bearing primary conidia **f, g** microconidia **h** villose spores. Scale bars: 10 mm (**a**); 20 μm (**b–h**).

#### Conidiobolus iuxtagenitus

Taxon classificationFungiEntomophthoralesAncylistaceae

S.D. Waters & Callaghan, Mycol. Res. 93(2): 223 (1989)

2BC62ED2-2D2F-5A03-82F3-B6D6572047FA

135617

Figure 5a–g

##### Specimens examined.

China, Anhui Province, Plant detritus, 8 Nov 2008, *C.F. Wang, RCEF 4445*.

##### Description.

Colonies on PDA at 21 °C after 3 d white, flat, slow-growing, reaching ca. 13 mm in diameter. Mycelia colourless, 5.5–11 μm wide. Primary conidiophores, positively phototropic, arising from hyphal segments, colourless, 28–75 × 7.5–10 μm, unbranched and producing a single globose conidium. Primary conidia forcibly discharged, globose, 27–37 × 21–28 μm, with a basal papilla 6–10 μm wide. Secondary conidia arising from primary conidia, similar to, but smaller than the primary ones, forcibly discharged. Tertiary conidium fusiform arising from primary conidia, 30–45 × 16–22 μm. Zygospores in a position separated by a short beak near a lateral conjugation, globose to subglobose, smooth, 21–25 × 17–24 μm, with a 1–2 μm thick wall.

##### Notes.

The ex-type living culture is ARSEF 6378 (United Kingdom, Staffordshire, Plant detritus, 31 October 1983, M. F. Smith).

**Figure 5.**
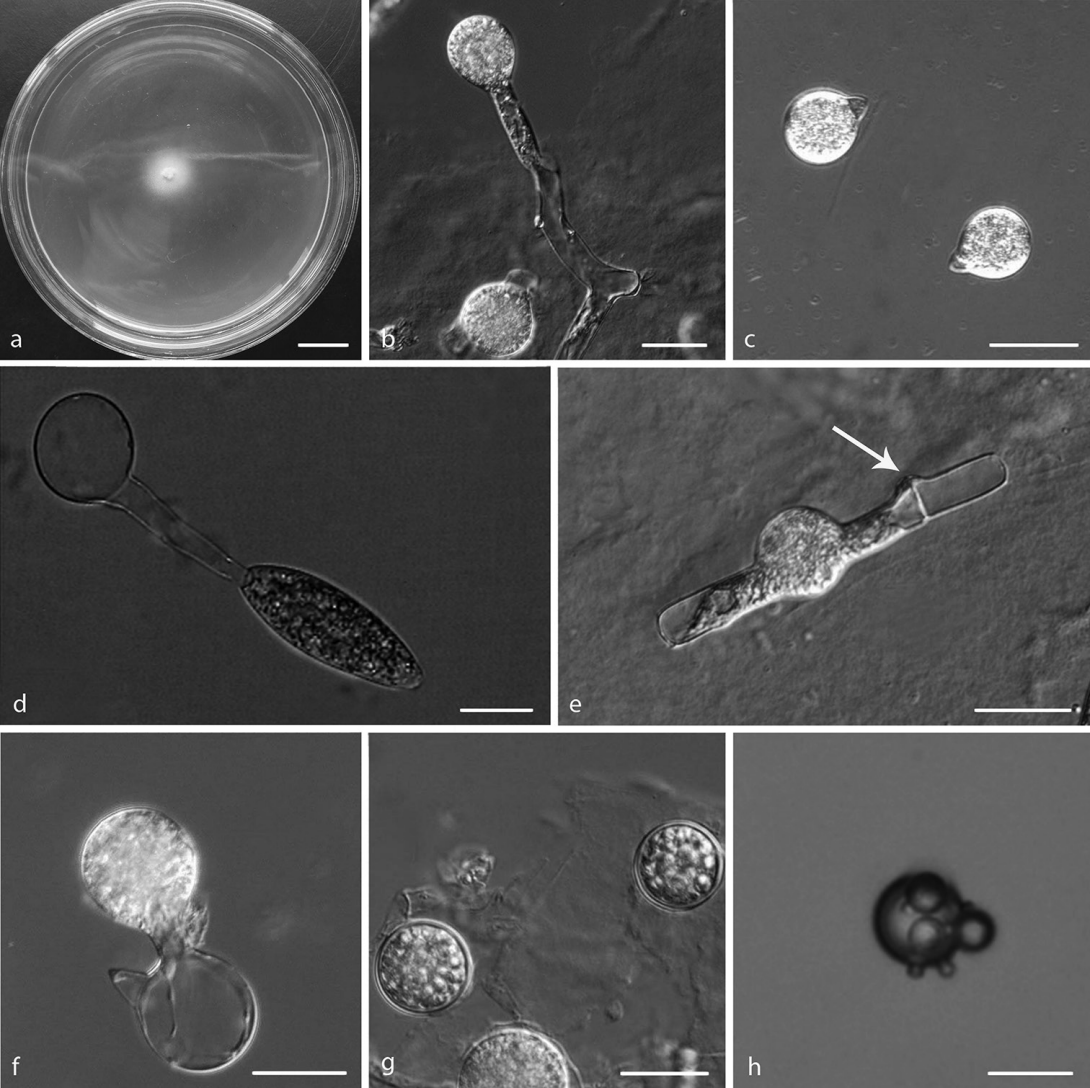
**a–g***Conidiobolus iuxtagenitus***h***Conidiobolus khandalensis***a** colony on PDA after 3 d at 21 °C **b** primary conidiophores bearing primary conidia **c** primary conidia **d** tertiary fusiform conidium from a globose spore **e** zygospore formation with the beak almost emptied of protoplasm **f** production of secondary conidia **g** zygospores **h** microconidia produced from global conidia. Scale bars: 10 mm (**a**); 20 μm (**b–h**).

#### Conidiobolus khandalensis

Taxon classificationFungiEntomophthoralesAncylistaceae

Sriniv. & Thirum., Mycologia 54(6): 692 (1963) [1962]

E009E85B-7E6F-59CA-A1AA-BC7893AB6585

328754

Figure 5h

##### Specimens examined.

India, Khandala, Dec. 1961, *Srinivasan and Thirumalachar, ATCC 15162*.

##### Description.

Refer to Srinivasan and Thirumalachar (1962b). Microconidia produced from global conidia on the 2% water-agar at 16 °C (Fig. 5h).

##### Notes.

According to the original morphological description (Srinivasan and Thirumalachar 1962b) and the re-examination by King (1977), microconidia have not been reported. However, we observed the microconidia produced from global conidia on 2% water-agar at 16 °C. Moreover, this specimen was located in the *Conidiobolus* lineage (Figure 1) which was supported by our morphological analyses.

#### Microconidiobolus

Taxon classificationFungiEntomophthoralesAncylistaceae

B. Huang & Y. Nie gen. nov.

744CDD7E-6E4B-56B4-BF10-59FE98E3F107

831597

##### Etymology.

Referring to small discharged primary conidia.

##### Type species.

*Microconidiobolus paulus* (Drechsler) B. Huang & Y. Nie.

##### Description.

Mycelia colourless. Primary conidiophores simple and short, bearing a single primary conidia. Primary conidia forcibly discharged, multinucleate, colourless, globose to obovoid, usually small, mostly less than 20 μm. Only globose replicative conidia produced, similar and smaller than primary conidia. Chlamydospores globose, formed terminally on hyphae or from globose cells by thickening of the wall. Zygospores formed in axial alignment with two conjugating segments, globose to ellipsoidal, smooth and yellowish.

##### Notes.

This genus includes three species producing smaller primary conidia (mostly less than 20 μm) without microspores or capilliconidia compared to other *Conidiobolus* spp. These three species are *C. nodosus*, *C. paulus* and *C. terrestris*. According to the taxonomic scheme of *Conidiobolus* by King (1977), *C. undulatus* is a synonym of *C. paulus*, which is supported herein by molecular evidence (Figure 1). However, the phylogeny does not support *C. nodosus* and *C. terrestris* as synonyms of *C. lachnodes*, since the former two were located in clade IV and the latter in clade II (Figure 1). Therefore, we accept the taxonomic status at species level for *C. nodosus* and *C. terrestris*, based on the morphological and phylogenetic analyses.

#### Microconidiobolus paulus

Taxon classificationFungiEntomophthoralesAncylistaceae

(Drechsler) B. Huang & Y. Nie comb. nov.

E2B29A52-D392-543D-A5BF-D98B97B87E44

831605

Conidiobolus paulus Drechsler, Bull. Torrey bot. Club. 84: 269 (1957). Basionym.
= Conidiobolus undulatus Drechsler, Bull. Torrey bot. Club. 84: 275 (1957).
= Conidiobolus parvus Drechsler, Bull. Torrey bot. Club. 89: 233 (1962).

##### Description.

Refer to Drechsler (1957a).

##### Notes.

The ex-type living culture is ATCC 12942 (United States, Wisconsin, 18 November 1954, Drechsler).

#### Neoconidiobolus

Taxon classificationFungiEntomophthoralesAncylistaceae

B. Huang & Y. Nie gen. nov.

8A004282-B8EF-516F-A99D-77EBD48BE926

831598

##### Etymology.

Referring to the subgenus Conidiobolus raised to generic rank.

##### Type species.

*Neoconidiobolus thromboides* (Drechsler) B. Huang & Y. Nie.

##### Description.

Mycelia colourless. Primary conidiophores simple, sometimes branched from hyphal knots or differentiated from aerial hyphae, positively phototropic, bearing a single primary conidium. Primary conidia forcibly discharged, multinucleate, colourless, globose, pyriform to obovoid. Replicative conidia similar and smaller than primary conidia. Chlamydospores globose, formed terminally on hyphae or from globose cells by thickening of the wall. Zygospores formed in axial alignment with two conjugating segments, globose to ellipsoidal, smooth, colourless, rarely pale yellowish.

##### Notes.

The genus Neoconidiobolus is strikingly similar to the subgenus Conidiobolus which produces neither microconidia nor capilliconidia. All members in the clade of *Neoconidiobolus* share the following characteristics: forcibly discharged, colourless, globose, pyriform to obovoid primary conidia. Two kinds of replicative conidia produced. One is discharged, similar and smaller than primary conidia and the other is elongate and forcibly discharged. Two types of resting spores produced: zygospores and chlamydospores.

#### Neoconidiobolus thromboides

Taxon classificationFungiEntomophthoralesAncylistaceae

(Drechsler) B. Huang & Y. Nie comb. nov.

672EE869-86FB-5516-BDC0-D34E25189680

831606

Figure 6

Conidiobolus thromboides Drechsler, J. Wash. Acad. Sci. 43: 38 (1953). Basionym.

##### Specimens examined.

China, Anhui Province, Plant detritus, 21 Feb 2009, *C.F. Wang, RCEF 4492*.

##### Description.

Colonies grown on PDA for 3 d at 25 °C, white, reaching ca. 30 mm diameter. Mycelium colourless, ﬁlamentous, 5–7.5 µm wide. Primary conidiophores colourless, unbranched and producing a single conidium, 50–122.5 × 6–16.5 µm. Primary conidia forcibly discharged, colourless, globose to subglobose, 20–26.5 µm wide, 26.5–34 µm long, including a basal papilla 6–10 µm wide. Secondary conidia globose, forming from the primary conidia. Zygospores most often formed between segments of separate hyphae. Mature zygospores smooth, globose to subglobose, 25–30 μm in diameter with wall 2–3 μm thick.

##### Notes.

The ex-type living culture is ATCC 12587 (United States, New Hampshire, September 1957, Drechsler).

**Figure 6.**
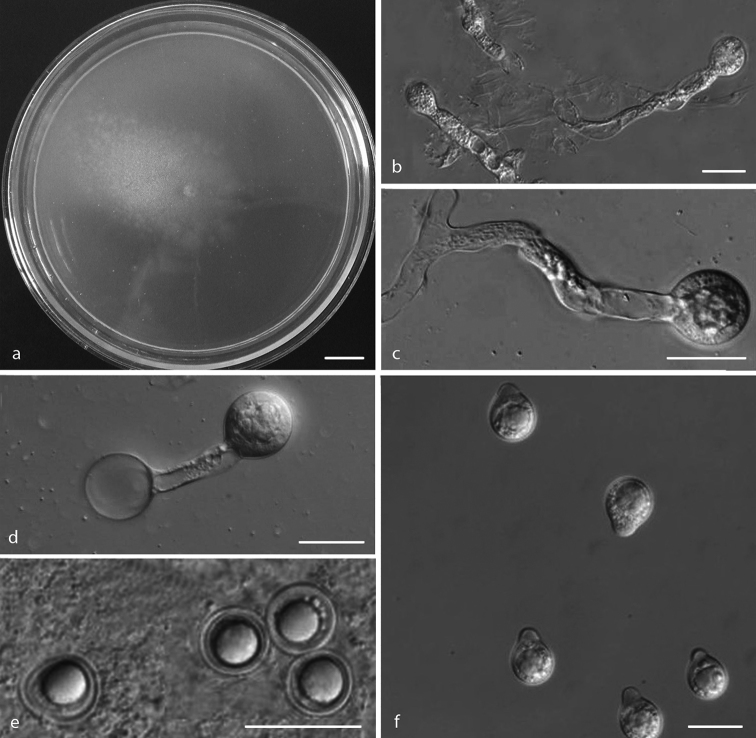
Neoconidiobolus thromboides**a** colony on PDA after 3 d at 25 °C **b, c** primary conidiophores bearing primary conidia **d** production of secondary conidia **e** zygospores **f** primary conidia. Scale bars: 10 mm (**a**); 20 μm (**b–d, f**); 40 μm (**e**).

### More new combinations

In addition to previously described taxa, more new combinations were proposed herein and their descriptions refer to relevant protologues.

#### Capillidium bangalorense

Taxon classificationFungiEntomophthoralesAncylistaceae

(Sriniv. & Thirum.) B. Huang & Y. Nie comb. nov.

49E94C4C-B2DA-5D26-98C6-CCA98B7576B6

831607

Conidiobolus bangalorensis Sriniv. & Thirum., Mycologia 59(4): 702 (1967). Basionym.

#### Capillidium denaeosporum

Taxon classificationFungiEntomophthoralesAncylistaceae

(Drechsler) B. Huang & Y. Nie comb. nov.

A9419A69-2648-5350-A992-B432913ECAD1

831608

Conidiobolus denaeosporus Drechsler, J. Wash. Acad. Sci. 47: 309 (1957). Basionym.

#### Capillidium lobatum

Taxon classificationFungiEntomophthoralesAncylistaceae

(Sriniv. & Thirum.) B. Huang & Y. Nie comb. nov.

63DB8CDB-AF6B-5281-8373-450ED3B2764B

831609

Conidiobolus lobatus Sriniv. & Thirum., J. Elisha Mitchell scient. Soc. 84: 212 (1968). Basionym.

#### Capillidium pumilum

Taxon classificationFungiEntomophthoralesAncylistaceae

(Drechsler) B. Huang & Y. Nie comb. nov.

098895D0-4C8A-590C-A70A-E436E6E987D9

831610

Conidiobolus pumilus Drechsler, J. Wash. Acad. Sci. 45: 115 (1955). Basionym.
= Conidiobolus globuliferus Drechsler, Am. J. Bot. 43: 783 (1957) [1956].
= Conidiobolus inordinatus Drechsler, J. Wash. Acad. Sci. 47: 312 (1957).

#### Capillidium rhysosporum

Taxon classificationFungiEntomophthoralesAncylistaceae

(Drechsler) B. Huang & Y. Nie comb. nov.

CDF80202-031E-5743-956C-E7B685CB859F

831611

Conidiobolus rhysosporus Drechsler, Am. J. Bot. 41: 567 (1954). Basionym.

#### Microconidiobolus nodosus

Taxon classificationFungiEntomophthoralesAncylistaceae

(Sriniv. & Thirum.) B. Huang & Y. Nie comb. nov.

0645FC92-B51F-5002-A8B6-0B825ACBE86D

831624

Conidiobolus nodosus Sriniv. & Thirum., Mycologia 59(4): 705 (1967). Basionym.

#### Microconidiobolus terrestris

Taxon classificationFungiEntomophthoralesAncylistaceae

(Sriniv. & Thirum.) B. Huang & Y. Nie comb. nov.

2F620080-2CCB-5A7C-897C-E4D1D3C2DCDE

831625

Conidiobolus terrestris Sriniv. & Thirum., Mycopathol. Mycol. appl. 36(3–4): 344 (1968). Basionym.

#### Neoconidiobolus couchii

Taxon classificationFungiEntomophthoralesAncylistaceae

(Sriniv. & Thirum.) B. Huang & Y. Nie comb. nov.

7C8ECD45-9291-5244-9F60-A808D89F7ECA

831626

Conidiobolus couchii Sriniv. & Thirum., J. Elisha Mitchell scient. Soc. 84: 211 (1968). Basionym.

#### Neoconidiobolus lachnodes

Taxon classificationFungiEntomophthoralesAncylistaceae

(Drechsler) B. Huang & Y. Nie comb. nov.

A8105E3E-8740-529B-AF8F-B699C93DBD05

831627

Conidiobolus lachnodes Drechsler, Am. J. Bot. 42: 442 (1955). Basionym.

#### Neoconidiobolus mirabilis

Taxon classificationFungiEntomophthoralesAncylistaceae

(Y. Nie & B. Huang) B. Huang & Y. Nie comb. nov.

6531CB5C-3134-596D-A5BB-EF4FF6757255

831628

Conidiobolus mirabilis Y. Nie & B. Huang, Mycol. Progr. 17(10): 1204 (2018). Basionym.

#### Neoconidiobolus osmodes

Taxon classificationFungiEntomophthoralesAncylistaceae

(Drechsler) B. Huang & Y. Nie comb. nov.

FEAA69F5-A749-5BB0-9095-AEF429D1BAFA

831629

Conidiobolus osmodes Drechsler, Am. J. Bot. 41: 571 (1954). Basionym.
= Conidiobolus antarcticus S. Tosi, Caretta & Humber, Mycotaxon 90(2): 344 (2004).

#### Neoconidiobolus pachyzygosporus

Taxon classificationFungiEntomophthoralesAncylistaceae

(Y. Nie & B. Huang) B. Huang & Y. Nie comb. nov.

50B684D1-0579-57E4-808E-4B1C906FDFEE

831630

Conidiobolus pachyzygosporus Y. Nie & B. Huang, Mycol. Progr. 17(10): 1206 (2018). Basionym.

#### Neoconidiobolus sinensis

Taxon classificationFungiEntomophthoralesAncylistaceae

(Y. Nie, X.Y. Liu & B. Huang) B. Huang & Y. Nie comb. nov.

278F1289-BCF8-5E30-B7FA-6090B1923C42

831631

Conidiobolus sinensis Y. Nie, X.Y. Liu & B. Huang, Mycotaxon 120: 432 (2012). Basionym.

#### Neoconidiobolus stilbeus

Taxon classificationFungiEntomophthoralesAncylistaceae

(Y. Nie & B. Huang) B. Huang & Y. Nie comb. nov.

A39E4A47-D4E5-532D-934E-D305571D2923

831632

Conidiobolus stilbeus Y. Nie & B. Huang, Mycosphere 7(6): 804 (2016). Basionym.

#### Neoconidiobolus stromoideus

Taxon classificationFungiEntomophthoralesAncylistaceae

(Sriniv. & Thirum.) B. Huang & Y. Nie comb. nov.

EBB4B068-7277-517B-B39B-9E1C0CA6D6FF

831633

Conidiobolus stromoideus Sriniv. & Thirum., Sydowia 16(1–6): 65 (1963) [1962]. Basionym.

#### Neoconidiobolus vermicola

Taxon classificationFungiEntomophthoralesAncylistaceae

(J.S. McCulloch) B. Huang & Y. Nie comb. nov.

0D7F7877-D73B-57D1-9B8E-5C0662E1290C

831634

Entomophthora vermicola J.S. McCulloch, Trans. Br. mycol. Soc. 68(2): 173 (1977). Basionym.
Macrobiotophthora vermicola (J.S. McCulloch) B.E. Tucker, Mycotaxon 13(3): 499 (1981).

## Discussion

The phylogenetic position of *Basidiobolus* in the Kingdom *Fungi* has been problematic for a long time. Previous phylogenetic analyses of the rDNA (18S, 28S and 5.8S) sequences grouped *Basidiobolus* outside or basal in the *Entomophthorales* (Nagahama et al. 1995; Jensen et al. 1998; White et al. 2006). Combined with the study of other protein-coding molecular markers, *Basidiobolus* was located inside the *Entomophthorales* (James et al. 2006). Recently, according to the phylogeny of much more available molecular data of entomophthoroid fungi in three families, *Basidiobolus* was grouped basal to other entomophthoroid taxa (Gryganskyi et al. 2012) which was also supported by the phylogenomic analyses of zygomycete fungi (Spatafora et al. 2016) and by the multi-gene analyses in this study. Although the morphological characteristics of *Batkoa* were similar to *Conidiobolus*, the *Batkoa* lineage appeared to be most closely related to the other taxa in the *Entomophthoraceae* Clade and should be distinguished from *Conidiobolus* lineage by its obligate pathogenicity for invertebrates and by staining readily, while most members of *Conidiobolus* are saprobic and non-staining.

The phylogenetic relationship of the genus *Conidiobolus* has been unclear for a long time, because of its high heterology (Gryganskyi et al. 2013). This article used more available ex-type strains to revise this genus, based on phylogeny and morphology. According to Figure 1, four main clades were reconstructed and the results showed that *Conidiobolus* s.l. is not a monophyletic group but paraphyletic with *Macrobiotophthora vermicola*. The *M. vermicola* was originally placed in *Entomophthora* (Mcculloch 1977) and transferred to *Macrobiotophthora*, based on the morphological characters of primary spores, secondary spores and zygospores (Tucker 1981). The paraphyletic relationship between *Macrobiotophthora vermicola* and *Conidiobolus* s.l. was also revealed by Gryganskyi et al. (2012). In this paper, we treated it as a new combination and, therefore, proposed a monophyletic group of the new genus *Neoconidiobolus*.

In Clade I of the genus *Capillidium*, seven species grouped in a monophyletic clade with good support (100/1.00) and the synapomorph of producing capilliconidia: *Conidiobolus adiaeretus* (= *Capillidium adiaeretum*), *Co. bangalorensis* (= *Ca. bangalorensis*), *Co. denaeosporus* (= *Ca. denaeosporum*), *Co. heterosporus* (= *Ca. heterosprum*), *Co. lobatus* (= *Ca. lobatum*), *Co. pumilus* (= *Ca. pumilum*) and *Co. rhysosporus* (= *Ca. rhysosporum*). As a note, *Co. denaeosporus* was synonymised with *Co. pumilus* (King 1976b), but herein its taxonomic status of species level was accepted according to the phylogeny. *Co. adiaeretus* forms not only capilliconidia but also microspores (Callaghan et al. 2000).

In Clade II of the genus *Neoconidiobolus*, all 14 strains comprising 10 species produce neither microspores nor capilliconidia. Amongst these, *C. antarcticus* was identified as a synonym of *C. osmodes* (Chen and Huang 2018), which was confirmed here as they grouped into a robust clade.

Considering its long history and significant impact, we kept and emended the genus *Conidiobolus* and the original illustrations of the type species *C. utriculosus* (Brefeld 1884) were designated as its lectotype. Thus, we were able to recognise clade III under the genus name *Conidiobolus* on the basis of its synapomorph, namely microspores. In Clade III of the genus *Conidiobolus*, all species definitely produce microspores, except *Conidiobolus dabieshanensis*, *C. iuxtagenitus*, *C. khandalensis* and *C. lichenicolus*. Microspores have never been observed in *C. dabieshanensis* and *C. iuxtagenitus* (King 1977; Waters and Callaghan 1989; Nie et al. 2017), but cases for *C. khandalensis* and *C. lichenicolus* are somewhat different. For *C. khandalensis*, the protologue did not document any microspores (Srinivasan and Thirumalachar 1962b; King 1977), but they can be observed on 2% water-agar at 16 °C (Fig. 5h). Although the microspore of *C. lichenicolus* was not mentioned in the original description, the ability to produce microspores has been exhibited in accordance with original illustrations (Srinivasan and Thirumalachar 1968a). The phylogeny also resulted in the following taxonomic treatments. On the one hand, some previously synonymised taxa recover their specific status, for example, *C. gonimodes*, *C. megalotocus* and *C. mycophagus* should be separated from *C. incongruus*, *C. macrosporus* and *C. mycophilus*, respectively. On the other hand, *C. chlamydosporus* is synonymised with *C. firmipilleus*.

In Clade IV of the genus *Microconidiobolus*, *Conidiobolus undulatus* was identified as a synonym of *C. paulus* (= *M. paulus*) by King (1976b), which is supported by our molecular data. Otherwise, *C. nodosus* (= *M. nodosus*) and *C. terrestris* (= *M. terristris*) were classified as synonyms of *C. lachnodes* (= *Neoconidiobolus lachnodes*) in the study of King (1976b). Morphologically, *C. lachnodes* bears larger primary conidia (9–25 × 10–27 μm) than *C. nodosus* (13–16 × 17–22 μm) and *C. terrestris* (8–12 μm in width) (Drechsler 1955b; Srinivasan and Thirumalachar 1967, 1968a). Furthermore, *C. lachnodes* was located in Clade II and is distantly related to *C. nodosus* and *C. terrestris*. Therefore, *C. nodosus* and *C. terrestris* are accepted as two distinct species. This clade comprises four ex-type strains, all producing smaller primary conidia (mostly less than 20 μm) and can be morphologically easily distinguished from other *Conidiobolus* species.

Phylogenetically, *Conidiobolus lamprauges* does group with Clade III and received strong bootstrap support (100/1.00). Morphologically, this species produces small primary conidia (12.5–20 × 15–22 μm) without microconidia or capilliconidia and is similar to species within Clade IV. Its taxonomic status remains unclear in the present study.

## Supplementary Material

XML Treatment for Capillidium

XML Treatment for Capillidium heterosporum

XML Treatment for Capillidium adiaeretum

XML Treatment for Conidiobolus

XML Treatment for Conidiobolus utriculosus

XML Treatment for Conidiobolus coronatus

XML Treatment for Conidiobolus iuxtagenitus

XML Treatment for Conidiobolus khandalensis

XML Treatment for Microconidiobolus

XML Treatment for Microconidiobolus paulus

XML Treatment for Neoconidiobolus

XML Treatment for Neoconidiobolus thromboides

XML Treatment for Capillidium bangalorense

XML Treatment for Capillidium denaeosporum

XML Treatment for Capillidium lobatum

XML Treatment for Capillidium pumilum

XML Treatment for Capillidium rhysosporum

XML Treatment for Microconidiobolus nodosus

XML Treatment for Microconidiobolus terrestris

XML Treatment for Neoconidiobolus couchii

XML Treatment for Neoconidiobolus lachnodes

XML Treatment for Neoconidiobolus mirabilis

XML Treatment for Neoconidiobolus osmodes

XML Treatment for Neoconidiobolus pachyzygosporus

XML Treatment for Neoconidiobolus sinensis

XML Treatment for Neoconidiobolus stilbeus

XML Treatment for Neoconidiobolus stromoideus

XML Treatment for Neoconidiobolus vermicola
